# Distribution of malaria patients seeking care in different types of health facilities during the implementation of National Malaria Elimination Programme

**DOI:** 10.1186/s12936-020-03205-9

**Published:** 2020-03-30

**Authors:** Gang Li, Donglan Zhang, Zhuo Chen, Da Feng, Xiaoyu Chen, Shangfeng Tang, Heejung Son, Zhenhua Wang, Yuanhang Xi, Zhanchun Feng

**Affiliations:** 1 School of Medicine and Health Management, Tongji Medical College, Huazhong University of Science and Technology, Wuhan, 430030 Hubei China; 2 Department of Health Policy and Management, College of Public Health, University of Georgia, Athens, GA 30602 USA; 3 School of Economics, University of Nottingham Ningbo China, Ningbo, 531200 Zhejiang China; 4 School of Pharmacy, Tongji Medical College, Huazhong University of Science and Technology, Wuhan, 430030 Hubei China; 5 Department of Mathematics, University of Georgia, Athens, GA 30602 USA

**Keywords:** Geographic variation, Care-seeking behavior, Geospatial distribution, Medical treatment preference

## Abstract

**Background:**

China launched the National Malaria Elimination Programme (NMEP) in 2010 and set the goal that all health facilities should be able to diagnose malaria. Additionally, hospitals at all levels could treat malaria by 2015. To provide a reference for the control of imported malaria, a study was conducted on the distribution of malaria patients seeking care in different types of health facilities.

**Methods:**

There were two data sources. One was obtained through the Infectious Diseases Information Reporting Management System (IDIRMS), which only contained the name of health facilities and the number of cases. The other was obtained through multistage stratified cluster sampling. Descriptive statistical analysis was used to investigate the distribution of malaria patients attending different types of health facilities (hospitals, township hospitals, and Centers for Disease Control and Prevention), hospital tiers (county-level, prefecture-level, and provincial-level), and hospital levels (primary, secondary, and tertiary). Chi-square test was also used to compare the proportions of patients seeking care outside their current residence region between different types of hospitals. Point maps were drawn to visualize the spatial distribution of hospitals reporting malaria cases, and flow maps were created to show the spatial flow of malaria patients by using the ArcGIS software.

**Results:**

The proportions of malaria patients who sought care in hospitals, township hospitals, and Centers for Disease Control and Prevention were 81.7%, 14.7%, and 3.6%, respectively. For those who sought care in hospitals, the percentages of patients who sought care in provincial-level, prefecture-level and county-level hospitals were 17.4%, 60.5% and 22.1%, correspondingly; the proportions of patients who sought care in tertiary hospitals, secondary hospitals, and primary hospitals were 59.8%, 39.9%, and 0.3%, respectively. Moreover, the proportions of patients seeking care in hospitals within county and prefectural administrative areas were 18.2%, 63.4%, respectively.

**Conclusion:**

During the implementation of NMEP, malaria patients tended to seek care in tertiary hospitals and prefecture-level hospitals, and more than half of patients could be treated in hospitals in prefecture-level areas. In the current phase, it is necessary to establish referral system from county-level hospitals to higher-level hospitals for malaria treatment.

## Background

Malaria is one of the most prevalent mosquito-borne infectious diseases in the world, which can have a damaging impact on society and the economy [1–3]. According to the latest available data, an estimated 228 million malaria cases occurred worldwide, and 405,000 deaths associated with malaria were reported in 2018 [4, 5]. Malaria had been prevalent since the foundation of the People’s Republic of China [6]. After the Chinese government put a great deal of efforts into the control of malaria epidemics, the morbidity and mortality rates of malaria had reached their lowest level historically in the late twentieth century [7]. However, after 2000, a malaria resurgence occurred in China, and peaked in 2006 with approximately 64,178 malaria cases reported [8–10]. China immediately launched the National Malaria Control Programme (2006–2015) with the partnership of the Global Fund project [11, 12]. With the effective implementation of measures, such as multi-sectorial cooperation and communication strategy, the incidence of autochthonous malaria cases had declined sharply during the period 2006–2010 in China [13–15].

After 2010, the epidemiology of malaria was different from that in the past. Almost all cases were imported cases due to the increasing number of immigrant workers and foreign travellers [16]. At the same time, the area where malaria transmission was occurring during this period in China had expanded from 24 traditional malaria-endemic provinces to 31 provinces across the country. Most patients suffering from malaria were low-literacy labourers from rural areas, and their awareness of being infected with malaria was low. Despite they were suffering from malaria, the initial symptoms of these patients were fever, chills, and sweating [17]. They always sought care at a grassroots medical institution nearby with symptoms of cold. Some previous studies also reported deaths from malaria patients who developed severe illnesses due to misdiagnosis by grassroots medical institutions [18, 19]. In 2010, in order to enable malaria patients to receive timely treatment within the county area and reduce delays, the Chinese government also had launched the National Malaria Elimination Programme (NMEP) (2010–2020) [3, 8, 20]. Through this action plan, the government had strengthened the training of malaria diagnosis and treatment for clinicians in different types of health facilities to achieve the goal that all health facilities had the ability to diagnose malaria along with the hospitals at all levels, which had the ability to treat malaria by 2015 [21, 22]. In China, the Centers for Disease Control and Prevention (CDCs) provide malaria diagnostic services and free anti-malarial medications; clinics, township hospitals, and hospitals at all levels perform both diagnostic and treatment services [23]. If the township hospital failed to treat malaria patients after making an exact diagnosis, patients would be referred to county-level or higher-level hospitals [24].

As a category B notifiable infectious disease in China, health facilities at all levels have the responsibility to report malaria cases within 24 h after diagnosis according to the law on the Prevention and Control of Infectious Diseases [25]. In a way, if patients were diagnosed as malaria, their visits to health facilities would be recorded. Some studies have also described the distribution of malaria patients seeking care in different types of health facilities based on the reporting data [26]. However, there are few studies focus on the proportions of malaria patients seeking care in hospitals within county-level or prefecture-level areas. To address this knowledge gap, this study describes the distribution of malaria patients in different types of health facilities, and the proportions of patients seeking care in hospitals within county and prefectural administrative area in the context of the eradication phase of malaria.

## Methods

### Brief profile of study area

**Fig. 1:**
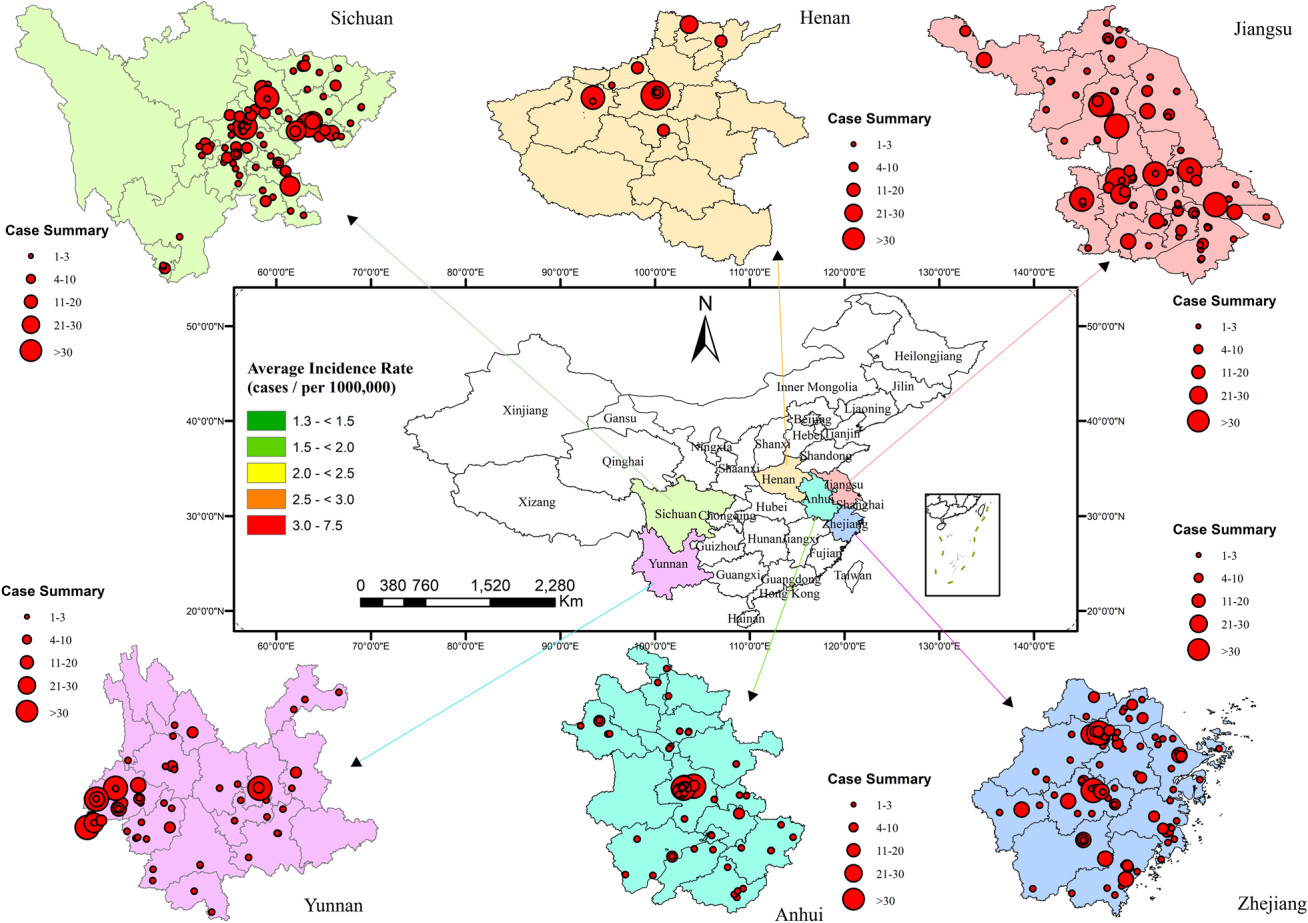
Spatial distribution of hospitals for malaria treatment

Two provinces with the highest incidence of malaria were selected in each region of China [11, 27], which were Zhejiang and Jiangsu provinces in Eastern China; Henan and Anhui provinces in Central China; Yunnan and Sichuan provinces in Western China (Fig. 1). The population of Zhejiang stands at 57 million, and Jiangsu is the fifth most populous and the most densely populated nationally during the 2019 census. Both of them have a humid subtropical climate. Anhui and Henan are landlocked provinces in Central China, with a population of 63 million and 96 million in 2019, respectively. Most of their cities have a continental monsoon humid climate. Sichuan adjoins the Tibetan Plateau in the west, with the third-largest population in China. Yunnan, a mountain and plateau region on the southwestern border of China, with a subtropical climate. Both Jiangsu and Zhejiang ranked top fifth in Gross Domestic Product (GDP), followed by Henan, Sichuan, and Anhui, while Yunnan’s economy ranked behind in 2019. Compared with the provinces of Western China, the provinces of Eastern and Central China have well-developed transportation [28]. During 2014–2016, the average annual malaria incidence rates in Zhejiang, Jiangsu, Anhui, Henan, Sichuan, and Yunnan were 3.1, 3.0, 1.9, 1.3, 2.5, and 7.5 per million population, respectively.

### Data source

**Fig. 2:**
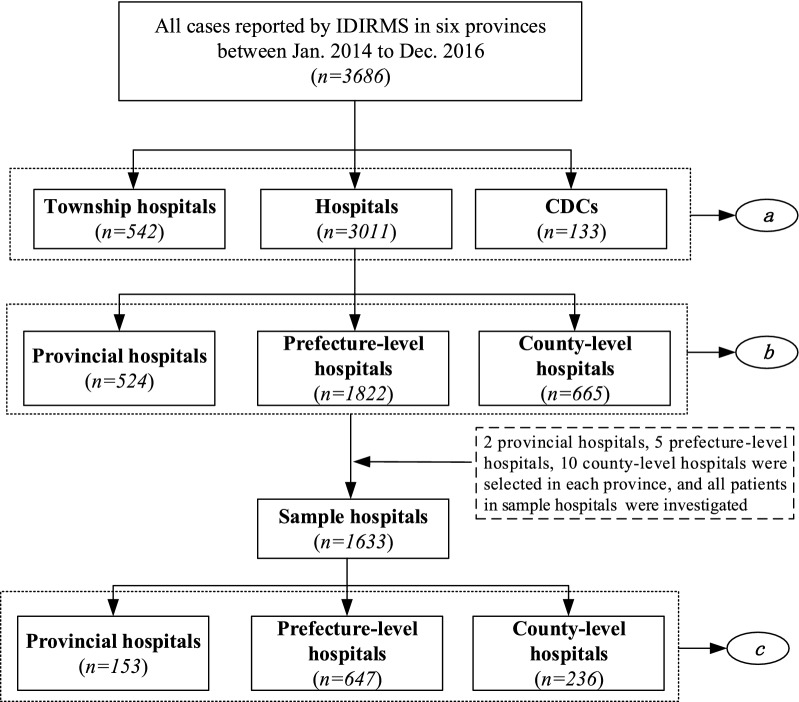
Study design and flow chart of the observations selection. The alphabet ***a*** represents that these data were used to analyse the distribution of malaria patients in different health facilities; the alphabet ***b*** represents that these data were used to analyse the distribution of malaria patients in different type hospitals; and the alphabet ***c*** represents that these data were used for spatial flow analysis of malaria patients who sought medical care in hospitals

There were two data sources. One was obtained through the Infectious Diseases Information Reporting Management System (IDIRMS) with the help of the National Health Commission of the People’s Republic of China. And the numbers of malaria cases reported by each health facility between January 2014 and December 2016 in sample provinces was collected, which was used to analyse the distribution of malaria patients in different types of health facilities. Due to the data obtained from IDIRMS lacking patients’ residential address information, it could not be used directly for the analysis of the proportions of patients seeking care in hospitals within county and prefectural administrative areas. The multistage stratified cluster sampling method was used to select two provincial-level hospitals, five prefecture-level hospitals, ten county-level hospitals with most malaria cases in each sample province. All patients’ medical records in each hospital selected were collected (Fig. 2). Variables collected from the medical record included patient demographics, patient residential address, hospital course. According to the number of cases reported in 102 hospitals selected, a total of 1868 cases from 102 hospitals should be investigated. In fact, we collected 1633 cases from 63 hospitals. The actual cases investigated accounted for 87.4% of cases reported in 102 selected hospitals. The actual cases investigated accounted for 54.2% of the total 3011 cases reported in all hospital. Except that Anhui Province had the most samples by default, samples from other provinces had a good representative.

### Variables and definition

The primary characteristics of hospitals (including address, hospital tier) were obtained from the National Medical Institution Inquiry System [29, 30]. The patient’s address was derived from the medical records. Each patient had two residences in the medical record, one was a permanent home residence, and the other was current residence. The current residence was used in this study, which usually referred to the place where the patients who had been living and working in the past 3 months, including the patients who were long-term migrants living and working in one location. The address used for analysis only needs to be accurate to the county level. So for the patients who lacked detailed address information, their local county government address was used to replace their current residence. Then the address was geocoded using Baidu Map Application Programming Interface (API).

**Fig. 3:**
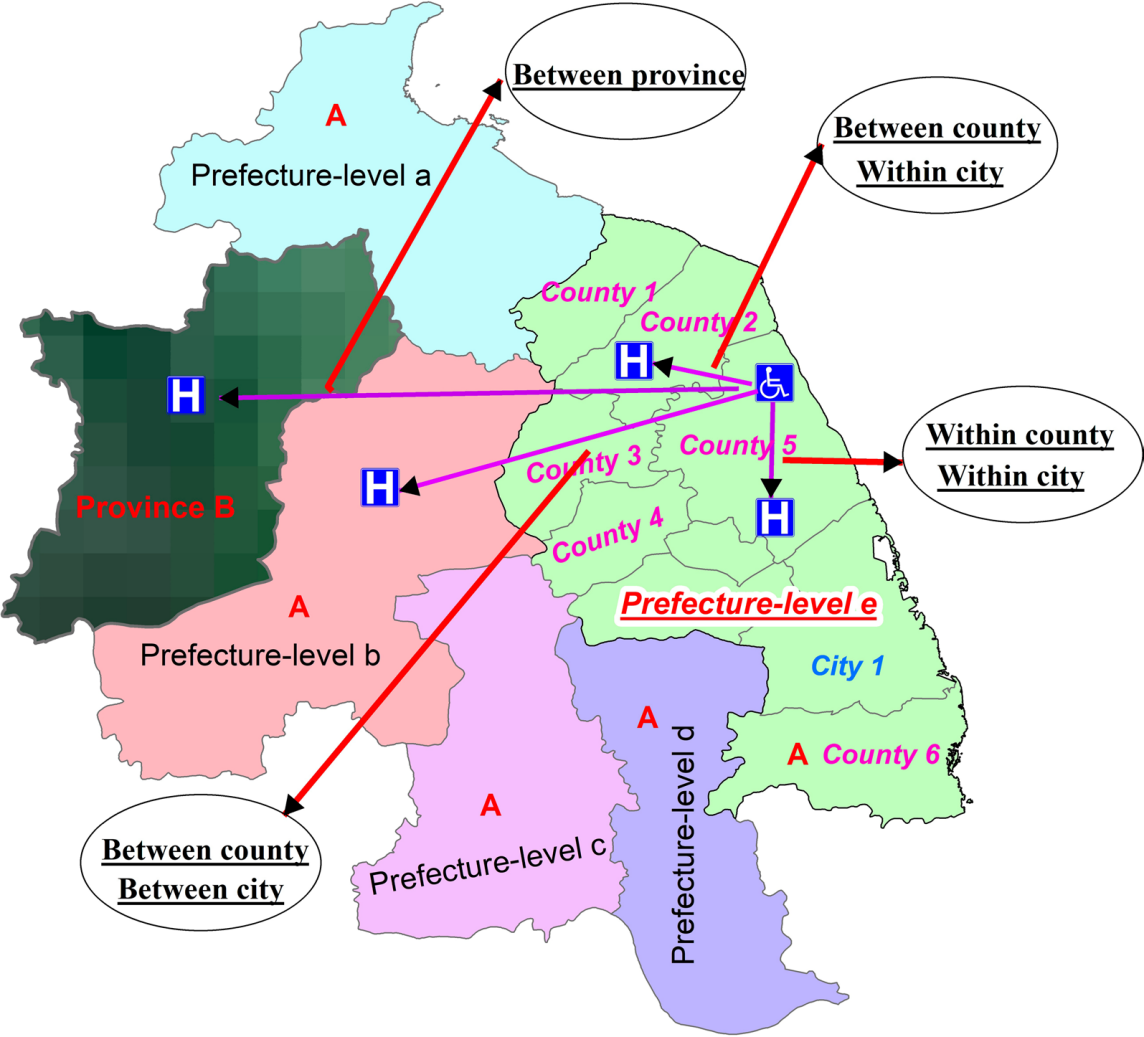
Illustration of terms related to patient flow

Within county patients meant that patients sought care within the same county as their current residence. Within city patients meant that patients sought care within the same prefecture-level area as their current residence. One province could be divided into several prefecture-level areas, and each prefecture-level area could be divided into one city and several counties in China. A more detailed terms description of within county and within city patients was shown in Fig. 3. The data collected was based on cases reported by health facilities in sample province. There may be some patients in the sample provinces seeking care in other provinces. In this study, it is assumed that the numbers of patients who flowed in was equal to the numbers of patients who flowed out. Patients from other provinces seeking care in the sample province were counted as patients in the sample province. And the proportion of within county patients was defined as, (a) *Proportion of within county patients in A province *=* Numbers of within county patients in A province/Total cases reported in A province*. The principle of calculating the proportion of within city patients, the proportion of within province patients was the same as mentioned above.

Hospital system in China was administrated by governments at the provincial-level, prefecture-level and county-level, so that hospitals can be classified into provincial-level hospitals, prefecture-level hospitals, and county-level hospitals. Hospitals of higher administrative level tend to have better medical resources. In a county-level region, clinics, township hospitals and county-level hospitals belong to grassroots medical institutions which provide basic medical services. Hospital levels are rated by hospital size and treatment level. Generally, the treatment level of the tertiary hospital is higher than that of the secondary hospital. Almost county-level hospitals belong to secondary hospitals, and most provincial-level hospitals and prefecture-level hospitals belong to tertiary hospitals in China. Usually provincial-level hospitals are located in the provincial capital city and prefecture-level hospitals are located in the city.

### Data analysis and mapping

Paper medical records were double entered into the Microsoft Excel 2016 (Microsoft Corporation, USA) and then were merged to electronic record data, and a descriptive analysis was conducted. Point maps were created to visualize the spatial distribution of hospitals reporting malaria cases using the ArcGIS software version 10.7 (ESRI, USA) [31]. Patient data was aggregated to county-level to describe the flow from different counties of residence to specific facilities. And flow maps were created to illustrate the patients’ spatial flow using the “*XY To Line*” analyst tool in ArcGIS.

### Ethical approval

The study was approved by the Ethics Committee of the Tongji Medical College of Huazhong University of Science and Technology (IORG0003571). Permission was granted by the National Health Commission of People’s Republic China and the manager of each hospital. Patient information was anonymized and de-identified before analysis.

## Results

### Distribution of malaria patients in different health facilities

A total of 3686 malaria patients sought care in 616 health facilities (township hospitals, hospitals, and CDCs) in six sample provinces during the period from 2014 to 2016. Overall, provinces in Western China had the largest number of malaria cases, followed by the Eastern and Central China.

The proportions of patients seeking care in hospitals, township hospitals, and CDCs were 81.7% (*n *= 3011), 14.7% (*n *= 542), and 3.6% (*n *= 133), respectively (Table 1). The proportions of patients seeking care in hospitals in most provinces had exceeded 90%, except for Zhejiang and Yunnan province. While the proportion of patients seeking care in township hospitals in Yunnan was over 40%, which was much higher than that in other provinces.

**Table 1:** Distribution of malaria patients seeking care in different types of health facilities

Type of medical institution	Total n = 3686	Eastern China		Central China		Western China	
		Zhejiang n = 517	Jiangsu n = 709	Anhui n = 341	Henan n = 432	Sichuan n = 622	Yunnan n = 1065
Hospitals	3011 (81.7%)	458 (88.6%)	667 (94.1%)	315 (92.4%)	421 (97.5%)	581 (93.4%)	569 (53.4%)
Township hospital	542 (14.7%)	13 (2.5%)	24 (3.4%)	22 (6.5%)	2 (0.46%)	20 (3.2%)	461 (43.3%)
CDCs	133 (3.6%)	46 (8.9%)	18 (2.5%)	4 (1.2%)	9 (2.08%)	21 (3.4%)	35 (3.3%)

### Distribution of malaria patients in different type hospitals

The proportions of patients seeking care in provincial-level, prefecture-level, and county-level hospitals were 17.4% (*n *= 524), 60.5% (*n *= 1822), and 22.1% (*n *= 665), respectively (Table 2). The proportions of patients seeking care in tertiary hospitals, secondary hospitals, and primary hospitals were 59.8% (*n *= 1801), 39.9% (*n *= 1202), and 0.3% (*n *= 8), respectively. The proportions of patients seeking care in tertiary hospitals in Zhejiang, Anhui, and Henan were relatively high, which were 73.1%, 69.2%, and 84.6%, respectively. The proportions of patients seeking care in provincial hospitals in Henan and Anhui were much higher than that in other provinces, which were 45.1% and 58.2%, respectively.

**Table 2:** Distribution of malaria patients seeking care in different types of hospitals

Hospital type	Total n = 3011	Eastern China		Central China		Western China	
		Zhejiang n = 458	Jiangsu n = 667	Anhui n = 315	Henan n = 421	Sichuan n = 581	Yunnan n = 569
Hospital tier							
Provincial-level	524 (17.4%)	65 (14.2%)	22 (3.3%)	142 (45.1%)	245 (58.2%)	43 (7.4%)	7 (1.2%)
Prefecture-level	1822 (60.5%)	279 (60.9%)	484 (72.6%)	87 (27.6%)	162 (38.5%)	396 (68.2%)	414 (72.8%)
County-level	665 (22.1%)	114 (24.9%)	161 (24.1%)	86 (27.3%)	14 (3.2%)	142 (24.4%)	148 (26.0%)
Hospital level							
Tertiary	1801 (59.8%)	335 (73.1%)	402 (60.3%)	218 (69.2%)	356 (84.6%)	329 (56.6%)	161 (28.3%)
Secondary	1202 (39.9%)	123 (26.9%)	258 (38.7%)	97 (30.8%)	65 (15.4%)	252 (43.4%)	407 (71.5%)
Primary	8 (0.3%)	0 (0.0%)	7 (1.0%)	0 (0.0%)	0 (0.0%)	0 (0.0%)	1 (0.2%)

### Geographical distribution of hospitals for treating malaria cases

Figure 1 shows the spatial distribution of hospitals reporting malaria cases, which included 3011 cases in 467 hospitals. The bigger dots represent large number of cases, and the smaller dots represent small number of cases treated in hospitals. It could be seen that the hospitals located in each provincial capital city treated most malaria cases. Hospitals located in a few certain cities in central and western China treated most cases. In a prefecture-level area, usually hospitals located in the city treated more cases than that in the county.

### Spatial flow analysis of malaria patients who sought medical care in hospitals

**Fig. 4:**
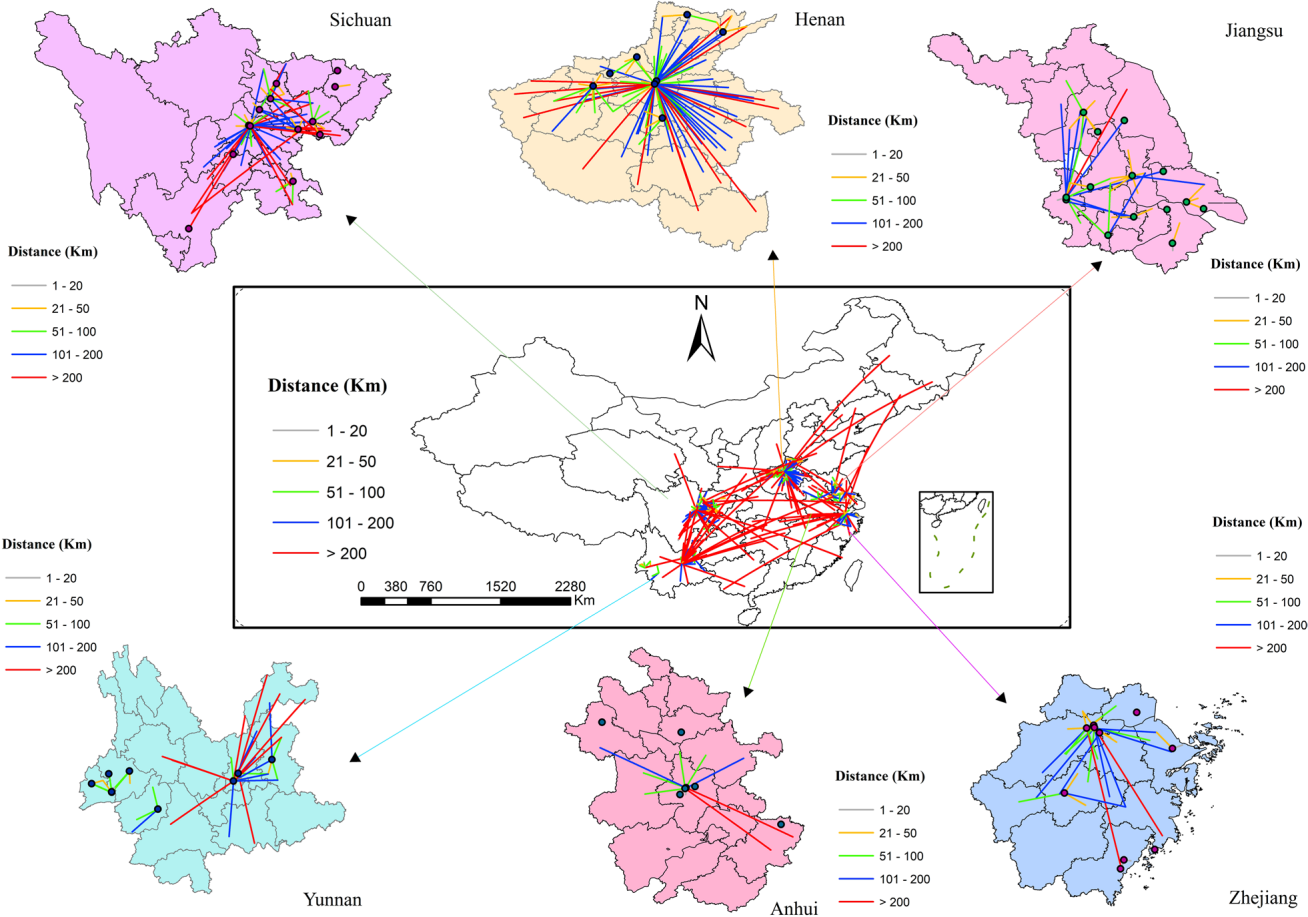
Flow maps of malaria patients seeking care from current residency to hospitals. Dots represent the location of hospitals where patients sought care, and starting points of the lines from dots represent the current residency of patients

Spatial flow refers to the path from a patient’s current residence to the hospital where he visited. According to the actual sampling results, 10 provincial hospitals, 25 prefecture-level hospitals, and 28 county-level hospitals were investigated, and a total of 1633 patients were surveyed. The proportions of patients seeking care in hospitals within provincial-level, prefecture-level, and county-level administrative areas were 96.1 (*n *= 1569), 63.4% (*n *= 1036), and 18.2% (*n *= 297), respectively (Table 3). The proportions of patients seeking care within county areas varied greatly among different provinces. Among these provinces, Jiangsu had the highest proportions of patients seeking care within county-level areas, at 47.7%. The proportions of patients seeking care within county-level areas in most provinces were less 10%. The proportions of patients seeking care within prefecture-level areas also varied significantly among different provinces. Among these provinces, Jiangsu had the highest proportions of patients seeking care within prefecture-level areas, at 90.9%. The proportions of patients seeking care within prefecture-level areas in most provinces were around 60%. Most patients from other prefecture-level areas sought care in the hospitals located in the provincial capital city in each province (Fig. 4). In each prefecture-level areas, most patients from other county-level areas sought care in the prefecture-level hospitals closer to where they lived.

**Table 3:** Characteristics of proportions of patients seeking care within county or within city in different types of hospitals

Province	Flow type	n	%	Hospital tier		P	Hospital level			P
				Secondary	Tertiary		Provincial	Prefecture	County	
Zhejiang	Within county	4	3.10	1 (10.0%)	3 (2.6%)	0.196	1 (1.9%)	0 (0.0%)	3 (33.3%)	0.510
n = 127	Within city	82	64.60	10 (100.0%)	72 (61.5%)	0.015	26 (50.0%)	48 (72.7%)	8 (88.9%)	0.011
Jiangsu	Within county	199	47.70	83 (92.2%)	116 (35.5%)	0.000	0 (0.0%)	53 (20.7%)	146 (96.7%)	0.000
n = 417	Within city	379	90.90	84 (93.3%)	295 (90.2%)	0.363	5 (50.0%)	228 (89.1%)	146 (96.7%)	0.000
Anhui	Within county	19	46.30	17 (65.4%)	2 (13.3%)	0.001	0 (0.0%)	0 (0.0%)	19 (100.0%)	0.000
n = 41	Within city	33	80.50	22 (84.6%)	11 (73.3%)	0.380	7 (70.0%)	7 (58.3%)	19 (100.0%)	0.011
Henan	Within county	38	9.00	21 (31.8%)	17 (4.8%)	0.000	15 (5.4%)	20 (14.6%)	3 (37.5%)	0.000
n = 421	Within city	224	53.20	65 (98.5%)	159 (44.8%)	0.000	87 (31.5%)	131 (95.6%)	6 (75.0%)	0.000
Sichuan	Within county	13	3.40	0 (0.0%)	13 (4.0%)	0.113	6 (19.4%)	7 (2.7%)	0 (0.0%)	0.000
n = 387	Within city	236	61.00	24 (39.3%)	212 (65.0%)	0.000	25 (80.6%)	158 (61.7%)	53 (53.0%)	0.021
Yunnan	Within county	24	10.00	4 (3.3%)	20 (16.8%)	0.000	1 (16.7%)	19 (16.7%)	4 (3.3%)	0.003
n = 240	Within city	82	34.20	13 (10.7%)	69 (58.0%)	0.000	3 (50.0%)	75 (65.8%)	4 (3.3%)	0.000
Total	Within county	297	18.20	126 (33.7%)	171 (13.6%)	0.000	23 (6.0%)	99 (11.8%)	175 (43.0%)	0.000
n = 1633	Within city	1036	63.40	218 (58.3%)	818 (65.0%)	0.018	153 (39.7%)	647 (76.9%)	236 (58%)	0.000

The meaning of ‘within’ designation was the same as the caption in Fig. 3

The proportions of patients seeking care within prefecture-level areas in secondary and tertiary hospitals were 41.7% (*n *= 156) and 35.0% (*n *= 441), respectively, which varied substantially from province to province. There was a significant difference in the proportions of patients seeking care within prefecture-level areas between the second and tertiary hospitals in Zhejiang, Henan, Sichuan, and Yunnan provinces.

## Discussion

This study presents the distribution of malaria patients seeking care in different types of health facilities and describes that most patients seeking care in provincial-level and prefecture-level hospitals were from areas where they lived far away from hospitals they visited. In this study, it was found that most malaria patients sought care in hospitals, followed by township hospitals and CDCs. This may be due to the fact that most patients were imported *Plasmodium falciparum* during post-stage of malaria elimination [32]. Patients with falciparum malaria were often in poor health, and needed timely medical first aid. Therefore, hospitals were playing an increasingly important role in the reporting, diagnosis, and treatment for them [33]. Compared with other provinces, the proportion of patients seeking care in township hospitals was slightly lower than the proportion of patients seeking care in hospitals in Yunnan, at 43.3%, which was much higher than that in other provinces. Yunnan province has international borders with Myanmar, Vietnam, and Laos, where malaria had been endemic. Most cases in Yunnan had been imported from these countries. The number of malaria cases in Yunnan once ranked first historically [34, 35]. Because malaria was a common disease in Yunnan, clinicians at local township hospitals had experience in treating malaria.

For patients seeking care in hospitals, it could be seen that proportions of patients seeking care in tertiary hospitals were much higher than any other two types of hospitals in most provinces except Yunnan province. One question was whether it was because these patients had more convenient access to tertiary hospitals that led to a higher proportion of that. It was found that the proportion of within county patients in tertiary hospitals in Yunnan was only 13.6%, which meant that most patients in tertiary hospitals came from other counties where far away from the tertiary hospitals. In China, each county generally has one or more county-level hospitals, and these hospitals usually belong to secondary hospitals. Why did they not go to the secondary hospital? There were maybe two reasons. One reason was that maybe clinicians in secondary hospitals failed to make an exact diagnosis as malaria, and then they referred patients to higher-level hospitals. Another reason was that patients might think that tertiary hospitals could provide better treatment services, and they would like to seek care in tertiary hospitals directly [36, 37]. The fact is that many patients would seek care in tertiary hospitals directly. However, the proportions of patients seeking care in hospitals within county-level areas were also low. It is speculated that maybe clinicians in secondary hospitals could not make a malaria diagnosis and treatment [24].

However, the proportions of patients seeking care within prefecture-level areas were relatively high in most provinces. It seemed that most malaria cases could be treated in prefecture-level areas. Compared with patients who sought care outside their prefecture-level areas, patients who sought care within prefecture-level areas would save time and prevent the delay and deterioration of the disease [38]. In the post-malaria eradication phase, it may be necessary to designate a hospital for malaria treatment in each prefecture-level area [39].

The characteristics of the distribution of patients seeking care in different types of hospitals between provinces varied greatly. As for Jiangsu province, both the proportions of patients seeking care within county-level areas and the proportions of patients seeking care within prefecture-level areas were much higher than that in other provinces. This meant that most malaria patients could be treated in hospitals within the prefecture-level and county-level areas, which was the goal that NMEP set. The measures taken by Jiangsu province to control the malaria epidemic were unique. The number of imported malaria cases in Jiangsu had been ranked third nationwide, and the number of falciparum malaria cases ranked first in the nation [40, 41]. The Jiangsu government put great importance on the elimination of malaria, and Jiangsu took the lead in putting forward the “1-3-7” approach: case reporting within 1 day, case investigation within 3 days, and case investigation and disposal of the epidemic within 7 days [42]. Meanwhile, various forms of technical training were carried out annually, and almost all staffs in various health facilities mastered the malaria Rapid Diagnostic Tests (RDTs) [43–45]. As a result, patients could be diagnosed and treated within county-level and prefecture-level areas.

## Limitations of the study

This study has at least three limitations. First, the number of cases reported by the sample hospitals and the location of the sample hospitals were considered in the selection of sample hospitals. Hospitals with a small number of reported malaria cases were not be prioritized for inclusion. Second, the actual sample cases were lower than expected due to the reluctance of sampled hospital staff or the loss of cases due to the renewal of the electronic case system. For example, only 41 cases were collected in Anhui Province, which would lead to unstable analysis results in Anhui. Third, this study focused on the hospitals reporting the cases. There may be some patients who went to other hospitals for medical services before they were diagnosed in the hospitals reporting the cases.

## Conclusions

This study described the distribution of malaria patients in different types of health facilities, and the proportions of patients seeking care in hospitals within county and prefectural administrative areas during the implementation of National Malaria Elimination Programme. During the implementation of the NMEP, malaria patients tended to seek care in tertiary hospitals and prefecture-level hospitals, and most patients could be treated in hospitals within prefecture-level areas. A lower proportion of patients seeking care within county-level areas reminded that the diagnosis and treatment capacity of county-level hospitals may be insufficient, which may lead to delays in malaria treatment, resulting in life-threatening and huge economic burden. In the elimination stage of malaria, the scope and extent of the malaria epidemic has fallen to its lowest level in history. Local governments should formulate corresponding prevention and control strategies according to their regional realities. It is necessary to continue to strengthen the monitoring of fever symptoms in people entering the epidemic areas [46], and consolidate the ability of clinicians in grassroots medical institutions to carry out RDTs and microscopic examination, and establish referral system from grassroots medical institutions to hospitals designated for malaria treatment.

## Data Availability

The datasets used in the current study are available from the corresponding author and will also be presented as an attachment in the article.

## Abbreviations

WHO: World Health Organization
NMEP: National Malaria Elimination Programme
RDTs: Rapid Diagnostic Tests
CDCs: Centers for Disease Control and Prevention
IDIRMS: Infectious Diseases Information Reporting Management System
GDP: Gross Domestic Product
